# Treatment of Localized Gingival Recession by Means Tunnel Technique after the Orthodontic Treatment. A Follow-Up of 1 Year

**DOI:** 10.1155/2020/8816510

**Published:** 2020-09-07

**Authors:** Marcelo Imano, Paula Porto Spada, Juliana Marchioro Souza Macalossi, Tatiana Miranda Deliberador

**Affiliations:** ^1^São Leopoldo Mandic and Private Pratice, Curitiba Paraná, Brazil; ^2^School of Health Sciences, Graduate Program in Dentistry, Universidade Positivo, Curitiba Paraná, Brazil; ^3^Periodontics and Professor of School of Health Sciences, Graduate Program in Dentistry, Universidade Positivo, Curitiba Paraná, Brazil

## Abstract

Facial and dental esthetics are the objectives of dental treatment, and, for this, it is necessary that specialties such as periodontics and orthodontics work together. The objective of this article is to report a clinical case with the solution of localized gingival recession after orthodontic treatment, using tunneled subepithelial connective tissue grafts with follow-up for 1 year. The patient underwent orthodontic treatment for 1 year. Prior to the start of treatment, a gingival recession of 5 mm was already present on tooth 31. One month after the completion of treatment, the patient was subjected to a tunneled subepithelial connective tissue graft, with the purpose of covering the exposed root. We observed the effectiveness of the procedure and patient satisfaction with the results obtained. The subepithelial connective tissue graft was successful in this case, and the collaboration of specialists is important to provide the best treatment for the patient.

## 1. Introduction

Orthodontic treatment may contribute significantly to the overall rehabilitation (esthetic and functional) of the stomatognathic system. Recent data show an increase of periodontal parameters after orthodontic treatment, indicating that it influences the accumulation and composition of the subgingival microbiota and subsequently induces more inflammation and higher bleeding on probing [1]. For this reason, periodontists and orthodontists have to work together to assess the effect of changes in incisor inclination owing to orthodontic treatment and the occurrence of gingival recession [2] and to decide when is best timing of soft tissue augmentation when a change in the inclination of the incisors is planned during orthodontic treatment [3].

Gingival/soft tissue recessions are defined as the displacement of the marginal tissue apical to the cementoenamel junction, and root exposure may lead to sensitivity and root caries and is not esthetically pleasing [4]. Gingival recession frequently occurs in the mandibular teeth, and the orthodontic movement of teeth, especially to positions outside the labial or lingual alveolar plate, could be a possible etiological factor for gingival recession [5]. Studies have been performed to evaluate the effects of orthodontic movement, especially those detected by the buccal movement of the incisors on the periodontium [6].

Lindht et al. [7] found that, according to clinical observation, gingival recession may occur during orthodontic treatment in areas with insufficient gingival tissue. In these cases, some authors suggest that the graft be performed to increase the gingival dimensions, before or after orthodontic treatment. Recent data from the literature do not allow to draw any conclusion on the best timing of soft tissue augmentation when a change in the inclination of the incisors is planned during orthodontic treatment [3].

Thus, the objective of this article is to report a clinical case with the solution to a localized gingival recession after the orthodontic treatment, using tunneled subepithelial connective tissue grafts with follow-up for 1 year.

## 2. Case Description

**Figure 1:**
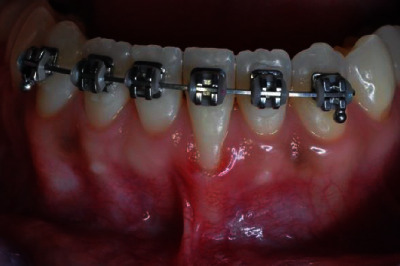
Intrabuccal images before removal of the orthodontic appliance.

The patient, S.N., who had a 5 mm gingival recession on the tooth 31 prior to the start of treatment, underwent orthodontic treatment for 1 year (Figure 1). After orthodontic therapy was completed, there was improvement in the occlusion and position of the teeth within the arch, including the lingualization of tooth 31. However, gingival recession still persisted. In order to recover the root, a tunneled subepithelial connective tissue graft was proposed to be conducted 1 month after orthodontic therapy had been completed.

Prior to the surgical procedure, an antisepsis was executed with povidone-iodine (PVPI), and the patient performed a mouthwash with 0.12% chlorhexidine digluconate for 1 minute. The surgical area was then anesthetized with 2% mepivacaine (MEPIADRE 100, DFL).

**Figure 2:**
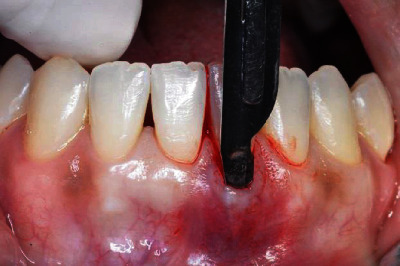
Intrasulcular incision.

**Figure 3:**
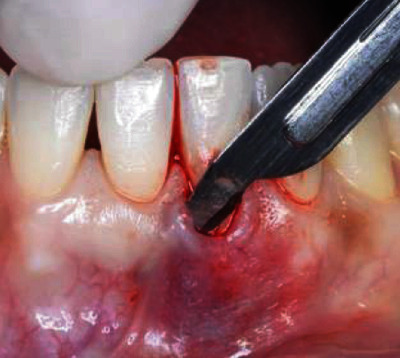
Intrasulcular incision preserving the interdental papillae.

**Figure 4:**
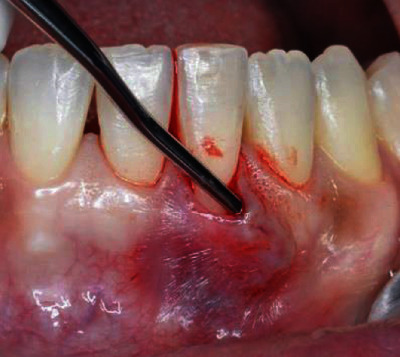
Detachment of the periosteal mucosa with no tensioned tissue.

**Figure 5:**
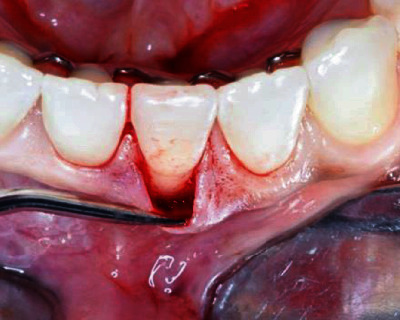
The tunnel patch created to receive the connective tissue graft.

The surgical procedure started with an intrasulcular incision (Figure 2), preserving the interdental papillae (Figure 3), followed by detachment of the periosteal mucosa through an incision with a No. 15c blade, so that the tissue was not under tension. During the detachment of the periosteal mucosa, the frenum was removed internally (Figure 4). A tunnel patch was then created to receive the connective graft (Figure 5).

**Figure 6:**
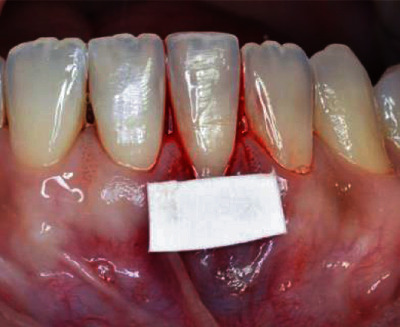
Surgical map with sterilized paper.

A surgical map with sterilized paper was performed, aiming at measuring the recipient area (Figure 6).

**Figure 7:**
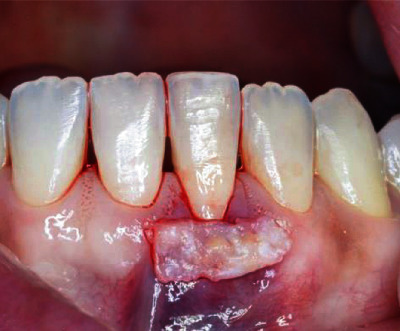
Graft positioned on the receiving area.

With the map, the connective tissue of the palate was removed in the region between teeth 14 and 16. The graft was positioned on the receiving area, in order to verify the location of the suture (Figure 7).

**Figure 8:**
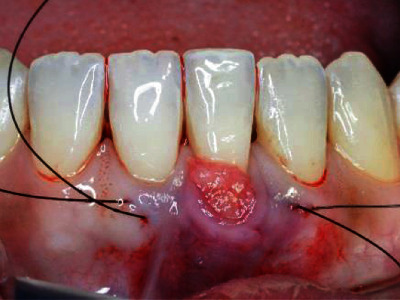
Two horizontal mattress sutures.

**Figure 9:**
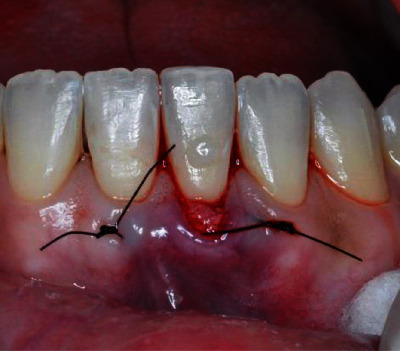
Sutures mesial and distal to the tooth.

Using 5.0 nylon thread, the graft was tunneled by two sutures (mesial and distal to the tooth) using the horizontal mattress technique (Figures 8 and 9).

**Figure 10:**
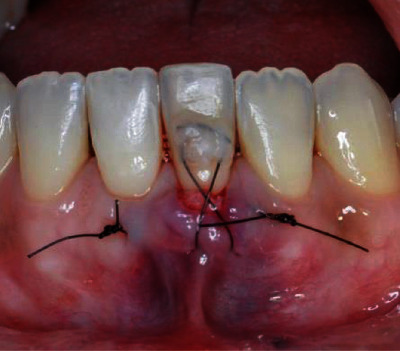
The suture Figure 8 with nonpolymerized resin.

In order to draw the flap to the coronal region, a suspensory suture was performed, used as coronal support to the composite resin polymerized in the crown of the tooth 31 (Figure 10).

**Figure 11:**
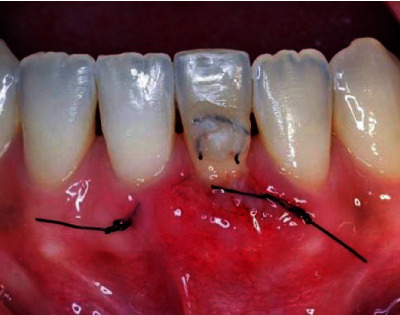
Removal of the coronal traction suture.

After 3 days, the coronal traction suture was removed to prevent the vestibular mucosa from being marked after complete healing (Figure 11).

**Figure 12:**
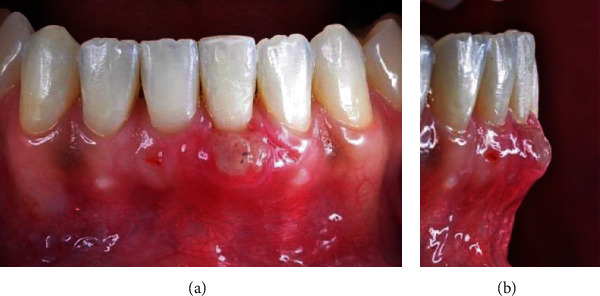
(a, b) Ten days postoperatively.

The sutures were removed 10 days postoperatively, and it was possible to observe the increase in volume in the vestibular region of the tooth 31 (Figures 12(a) and 12(b)).

**Figure 13:**
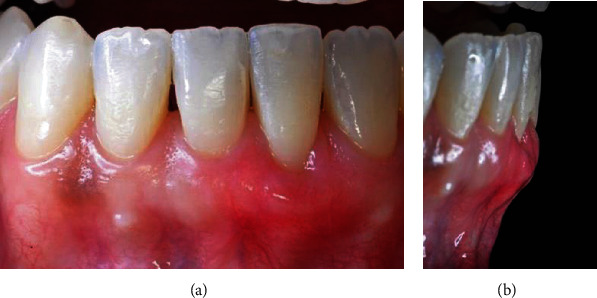
(a, b) 45 days postoperatively.

**Figure 14:**
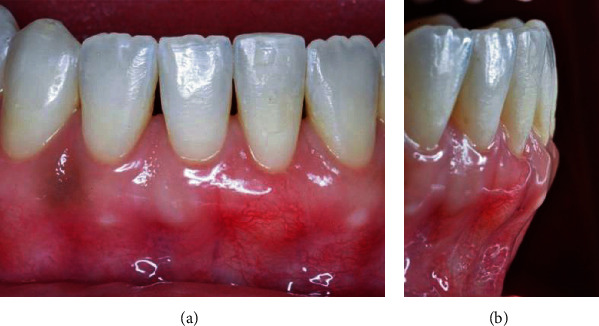
(a, b) 60 days postoperatively.

**Figure 15:**
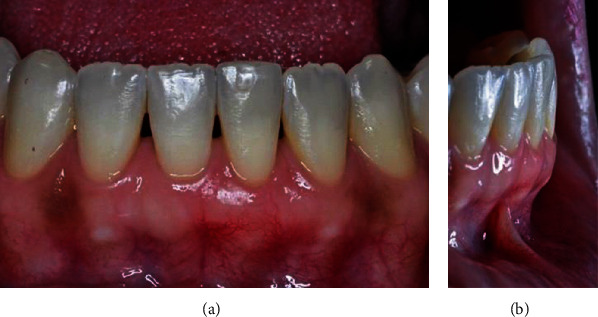
(a, b) Six months postoperatively.

**Figure 16:**
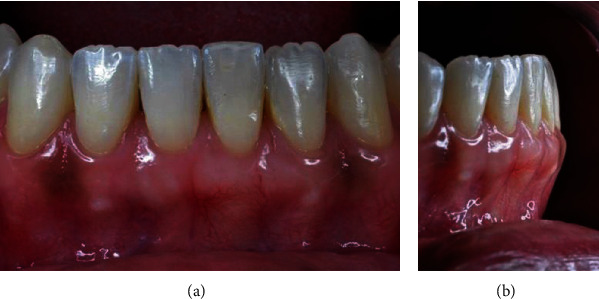
(a, b) One year postoperatively.

During the 45-day follow-up, we observed good cicatrization, root coverage, and an increase in gingival tissue volume in the vestibular region (Figures 13(a) and 13(b)). The patient was carried out after 60 days and was satisfied with the result found (Figures 14(a) and 14(b)), 6 months (Figures 15(a) and 15(b)), and one year (Figures 16(a) and 16(b)) after the surgery, during which we observed the formation of a keratinized gingiva and the complete root recover, besides the esthetic satisfaction reported by the patient.

## 3. Discussion

In 1992, Loe et al. [8] discovered gingival recession in more than 60% of the younger population (i.e., up to 20 years of age) and in more than 90% of the older population (>50 years) in a Western European population who received regular dental care.

Dorfman [9] indicated that mandibular incisors are the most likely to demonstrate this type of pathologic recession because the tooth-arch relationship results in labially prominent teeth covered with a thin or nonexistent labial plate of bone and inadequate or absent keratinized gingiva. In this case, tooth 31 had a root vestibular inclination prior to the orthodontic treatment and also presented a thin periodontal biotype and lower lip brake presence with papillary insertion leading to the development of gingival recession. The presence of gingival recession was observed before orthodontic movement, and it remained stable until the end of the treatment, and only then did we choose to perform the root covering surgical procedure to treat gingival recession.

Different surgical techniques have been introduced to treat gingival recession, including those involving autogenous tissue grafting (free gingival graft and connective tissue graft), various flap design, and guided tissue regeneration. A connective tissue graft provides the best probability of achieving complete root coverage with the greatest esthetic outcomes and probability of gingival margin stability [10]. Knowing the cause, prognosis, and the better time to treatment of gingival recession is the key to offer the best solution for the patient.

The surgical technique considered the gold standard for fine periodontal biotype is the connective tissue graft [10]. Nowadays, the quest and demand for esthetics has been advocated, and changes in the making of the flap are being described. Tunnel technique has recently gained popularity among clinicians for its promising clinical and esthetic results when treating gingival recessions (GRs) defects. [11].

The use of the tunnel technique also helps maintain adequate blood supply to the underlying graft and provides excellent adaptation of the graft to the recipient site [12]. The tunnel technique was found to be a highly effective procedure in treating gingival recession defects, exhibiting an overall mean root coverage of 82.8% for single and 87.9% for multiple gingival recession defects [11]. This technique has been increasingly used as a successful treatment option, as reported in this clinical case. The collaboration of specialists is important to provide the best treatment for the patient. Furthermore, we observed that the tunneled subepithelial connective tissue graft was successful in this case. The performance of the treatment of gingival recession after orthodontics was adequate and met the expectations of the patient.
